# The effect of long-haul COVID-19 toward domains of the health-related quality of life among recovered hospitalized patients

**DOI:** 10.3389/fpubh.2023.1068127

**Published:** 2023-08-03

**Authors:** Indang Trihandini, Manendra Muhtar, Dea Allan Karunia Sakti, Chintya Putri Erlianti

**Affiliations:** ^1^Biostatistics and Population Studies Department, Faculty of Public Health, Universitas Indonesia, Depok, Indonesia; ^2^Student of Master Program, Faculty of Public Health, Universitas Indonesia, Depok, Indonesia; ^3^Student of Undergraduate Studies, Faculty of Public Health, Universitas Indonesia, Depok, Indonesia

**Keywords:** long-haul COVID-19, SARS-CoV-2, domains of quality of life, WHOQOL-BREF, the health-related quality of life

## Abstract

**Background:**

People with long-haul COVID-19 could experience various health problems, from mild to severe. This research aimed to identify the effect of long-haul COVID-19, specifically on the Quality-of-Life domains experienced by COVID-19 patients who have been discharged.

**Methods:**

Data collection was done online, using data from DKI Jakarta hospitalized patients confirmed with and recovered from SARS-CoV-2 infections. We selected patients who have a minimum of 28 days after being hospitalized for COVID-19 positive. The Logistic regression technique was used to analyze the data. The questionnaire used in this research contained questions regarding long-haul COVID-19 symptoms and domains of Quality of Life, which WHOQOL-BREF measured. Before collecting data, we tested the questionnaire with 30 recovered patients hospitalized outside DKI Jakarta.

**Results:**

172 recovered inpatients who filled out the questionnaire correctly and were aged 18 years and above were randomly selected. Almost one-third (30.2%) of the recovered inpatients had long-haul COVID-19, with 23.8% experiencing one long-haul symptom and 6.4% experiencing more than one symptom. This research also showed that the long-haul effects of COVID-19 affected almost all domains of Quality of Life except the environmental one. Age, gender, and marital status were covariates for the association between long-haul COVID-19 and The Quality of Life.

**Conclusion:**

Continuing health services after the patient is discharged from the hospital is an important program for COVID-19 survivors because it can prevent a decline in the Quality of Life among patients due to the long-haul COVID-19.

## Introduction

Long-Haul COVID, also known as a post-COVID-19 syndrome, post-acute sequelae of COVID-19 (PASC), or chronic COVID syndrome (CCS), is a condition characterized by long-haul term sequelae appearing or persisting after the typical convalescence period of COVID-19 (1). Long-haul COVID can affect nearly every organ system with a wide range of symptoms commonly discussed, including fatigue, headaches, shortness of breath, anosmia, parosmia, muscle weakness, low fever, and cognitive dysfunction. Sequelae, nervous system and neurocognitive disorders, mental health disorders, metabolic disorders, cardiovascular disorders, gastrointestinal disorders, malaise, fatigue, musculoskeletal pain, and anemia (2–6). Italian researchers have studied 143 COVID-19 patients with the most severe symptoms; 60 days after the end of the disease, more than half of the patients still had bothersome symptoms, and 41% reported a worsening quality of life (7). In addition, Irish researchers reported that 128 patients with SARS-CoV-2 infection with PCR, on average 10 weeks after the initial symptoms of COVID-19, 52% reported persistent fatigue and 31% had not returned to work (8).

Long-haul COVID-19 tends to occur more frequently in survivors who have more severe symptoms during infection (9). The effectiveness of treatment measures to treat this condition is still being sought. These conditions associated with health-related quality of life have become an important issue in medical and psychological research. WHO defines the quality of life as individuals’ perceptions of their position in life in the context of the culture and value systems in which they live, as well as concerning their goals, expectations, standards, and concerns (10). WHO has assessed the quality of life through the World Health Quality of Life, abbreviated as WHQOL. The WHOQOL Group developed the WHOQOL with 15 international field centers simultaneously to develop a cross-culturally applicable quality of life assessment. The WHOQOL instrument consists of 100 questions, known as the WHOQOL-100. The WHOQOL-100 questionnaire, a multidimensional evaluation of individuals’ perceptions of their health status, psychosocial status, and other aspects of their lives (11). Subsequently, WHO created a simplified version of WHOQOL-100 called the WHOQOL-BREF (12). The WHOQOL-BREF questionnaire can be completed independently by the respondent or with the assistance of a trained interviewer (10, 12).

WHOQOL-BREF demonstrated good discriminant validity, content validity, internal consistency, and retest reliability and was highly correlated with WHOQOL-100 domain scores (12–14). The Indonesian version of the WHOQOL-BREF instrument showed a near-perfect match of the two general items and good agreement of the four domains. Therefore, it can be concluded that the WHOQOL-BREF is a consistent and stable instrument to measure the quality of life of Indonesian people in general (15–17).

Therefore, further in-depth studies are needed to examine the health problems in COVID-19 patients who have been declared cured after being discharged from the hospital. Thus, this research has aimed to identify the effect of Long-Haul COVID-19 symptoms on the Quality of Life of Recovered Patients in Hospitals in Jakarta. It is expected that the results of this research could be used to strengthen post-hospitalization supervision for recovered patients.

## Research method

The research related to the long-haul COVID-19 had passed an ethical permit and data collection permit from the DKI Health Office for 6 months. The research was conducted in DKI Jakarta, the capital of Indonesia and the center of the COVID-19 spread in Indonesia. Due to conditions that did not allow the data to be collected directly, data collection was conducted online using a database from reports of COVID-19 patients from various hospitals and health centers. The respondents were randomly selected from a list of COVID-19 patients who had recovered. This list was provided by the Provincial Health Office of DKI Jakarta. Several difficulties, such as a slow pace of data collection, as some patients who had recovered from COVID-19 refused to fill out online surveys, were encountered during the implementation. Eventually, after data cleaning and ensuring that the respondents met the inclusion criteria, (aged 18 or more, were hospitalized patients, and were interviewed a minimum of 28 days after being hospitalized COVID-19 positive), the total number of respondents who correctly filled out the form was 172.

There are many terms to describe the symptoms of Long-Haul COVID-19 and how to assess them. Long-Haul COVID-19, as the leading independent variable in this study, followed the term with a history of confirmed SARS-CoV-2 infection at an interview at least 28 days after completion of hospitalization for COVID-19 positive patients with symptoms who did not have the experience before becoming sick. Other independent variables, including gender, age, length of treatment, length from onset to interview, marital status, occupation, and respirator use, were determined as covariates. The WHOQOL-BREF was utilized to measure the dependent variable of this research: Quality of Life.

The four significant domains assessed in the WHOQOL-BREF can be described as (1) Physical Domain Score with seven items, (2) Psychological health with six items, (3) Social relationships with three items (Personal relationships; Sexual activity; and Social support) and (4) Environmental health with eight items. Each domain was separately analyzed using multivariate logistic regression.

## Results

Based on data from 172 participants in this research, the mean length of treatment (days) was 15.17 ± 8, while the mean length of the gap from baseline to interview (days) was 109.22 ± 69.71. Out of four quality-of-life domains, the lowest mean score was identified in the physical domain with 69.31 ± 12.31, while the highest score was indicated in the social domain with a mean of 78.29 ± 16.08. The mean score of the psychological and environmental domains was 74.89 ± 11.70 and 73.60 ± 13.30, respectively (Table 1).

**Table 1:** Descriptive statistics of length of treatment, length from onset to interview, quality of life domains.

Variable	Mean	SD	Min.	Max.
Length of Treatment (day)	15,17	8,00	7	42
Length from Onset to Interview (day)	109,22	69,72	29	309
WHOQOL Physical Domain Score	69,31	12,31	35,71	100
WHOQOL Psychological Domain Score	74,44	11,60	45,83	100
WHOQOL Social Domain Score	78,29	16,08	16,67	100
WHOQOL Environmental Domain Score	73,62	13,53	43,75	100

### Demographic characteristics

Table 2 describes the demographic characteristics of participants in this research. The proportion of males (48.3%) and females (51.7%) in this research tended to be equal. Most participants were aged 18–39 years (58.1%), married or living with partners (66.9%), and working in the private sector (55.2%). Out of 172 participants, only 20 (11.6%) used respiratory aids during their COVID-19 treatment. Among all participants, almost one-third (30.2%) experienced long-haul COVID-19 symptoms, with 23.8% having one and 6.4% having two or more symptoms.

**Table 2:** The demographic characteristics, history of treatments and the long-haul COVID 19.

No	Variables	Frequencies (%)
1	Gender
	Male	83 (48.3%)
	Female	89 (51.7%)
2	Age group
	18–39 years old	100 (58.1%)
	≥ 40 years old	72 (41.9%)
3	Marital status
	Married/Living with partner	115 (66.9%)
	Divorced/Single	57 (33.1%)
4	Occupation
	Civil servant & police & army	41 (23.8%)
	Private sector	95 (55.2%)
	Unemployed	36 (20.9%)
5	Length of treatment
	≤ 13 days (<2 weeks)	71 (41.3%)
	≥ 14 days (≥2 weeks)	101 (58.7%)
6	Respiratory used
	No	152 (88.4%)
	Yes	20 (11.6%)
7	Length from onset (to interview)
	≤ 3 months	92 (53.5%)
	>3 months	80 (46.5%)
8	Long-Haul COVID 19
	No	120 (69.8%)
	Yes	52 (30.2%)
9	Number of long-Haul COVID 19
	No	120 (69.8%)
	1 Symptom	41 (23.8%)
	> 1 Symptom	11 (6.4%)

Further information on long-haul COVID-19 is seen in Table 3. Eleven (11) symptoms that were collected, most of the participants experienced fatigue (16.3%), chest pain (7%), coughing (4.1%), breathing trouble (2.9%), as well as digestive disorder and headache (2.3%). One to two participants experienced other symptoms, such as ageusia, anosmia, memory loss, nausea, and joint pain.

**Table 3:** The list of the Long-Haul COVID 19 symptoms.

No	Variables	Frequencies (%)
1	Coughing
	No	165 (95.9%)
	Yes	7 (4.1%)
2	Digestive disorder
	No	168 (97.7%)
	Yes	4 (2.3%)
3	Ageusia
	No	169 (98.3%)
	Yes	3 (1.7%)
4	Anosmia
	No	170 (98.8%)
	Yes	2 (1.2%)
5	Headache
	No	168 (97.7%)
	Yes	4 (2.3%)
6	Memory loss
	No	171 (99.4%)
	Yes	1 (0.6%)
7	Nausea
	No	170 (98.8%)
	Yes	2 (1.2%)
8	Joint Pain
	No	171 (99.4%)
	Yes	1 (0.6%)
9	Fatigue
	No	144 (83.7%)
	Yes	28 (16.3%)
10	Breathing trouble
	No	167 (97.1%)
	Yes	5 (2.9%)
11	Chest pain
	No	160 (93.0%)
	Yes	12 (7.0%)

### Quality of life domains

Table 4 compares the average scores for each quality-of-life domain according to socio-demography, treatment history, and Long-Haul COVID-19 symptoms. The Comparison of the average of scores domains in Quality of life can be described that there was a difference in the average score of the social domain male higher rather than female groups (value of *p* 0.03). In the 18–39-year-old age group, the average psychological domain score was higher than the 40-year-old age group or older (*p* value 0.03). On the marital status of the participants, those who were married or living with a partner had a higher average psychological domain score than those from the single or divorced group (*p* value 0.007). On the variable duration of treatment, there was no significant difference in the average in all domains of Quality of Life. However, regarding the use of breathing during COVID-19 treatment, the two Quality of Life domains (Psychological and Environmental) have a significant average difference with *p* values of 0.01 and 0.02, respectively. The other variables were not proven to be significant.

**Table 4:** The quality of life domain scores for sociodemographic and health-related predictors.

Variables	Frequencies (%)	Mean ± SD															
		Physical domain score				Psychological domain score				Social domain score				Environmental domain score			
		Score	95% CI		*p* value	Score	95% CI		*p* value	Score	95% CI		*p* value	Score	95% CI		*p* value
			Lower	Upper			Lower	Upper			Lower	Upper			Lower	Upper	
Gender
Male	83 (48.3%)	70.22 ± 11.99	67.60	72.84	0.35	74.99 ± 12.03	72.37	77.63	0.54	81.02 ± 15.30	77.68	84.37	0.03*	74.96 ± 12.67	72.20	77.73	0.21
Female	89 (51.7%)	68.46 ± 12.61	65.80	71.11		73.92 ± 11.23	71.56	76.29		75.75 ± 16.46	72.28	79.22		72.37 ± 14.23	69.37	75.37	
Age group
18–39 years old	100 (58.1%)	70.86 ± 12.56	68.37	73.35	0.052	76.04 ± 11.41	73.78	78.31	0.03*	79.25 ± 16.65	75.95	82.55	0.36	73.72 ± 13.88	70.97	76.48	0.91
≥ 40 years old	72 (41.9%)	67.16 ± 11.71	64.41	69.91		72.22 ± 11.58	69.50	74.94		79.97 ± 15.29	73.37	80.56		73.48 ± 13.11	70.40	76.56	
Marital status
Married/Living with partner	115 (66.9%)	68.91 ± 11.59	66.77	71.05	0.55	73.26 ± 10.99	71.23	75.29	0.06	80.87 ± 13.87	78.31	83.43	0.007*	73.45 ± 12.74	71.09	75.81	0.82
Divorced/Single	57 (33.1%)	70.11 ± 13.73	66.47	73.75		76.83 ± 12.50	73.51	80.14		73.10 ± 18.89	68.08	78.11		73.96 ± 15.11	69.95	77.97	
Occupation
Civil servant & Police & army	41 (23.8%)	68.90 ± 12.81	64.86	72.95	0.51	73.88 ± 10.91	70.44	77.32	0.94	81.71 ± 14.34	77.18	86.23	0.30	75.54 ± 13.96	71.13	79.94	0.44
Private Sector	95 (55.2%)	70.19 ± 11.39	67.87	72.51		74.65 ± 11.07	72.39	76.90		77.19 ± 15.95	73.94	80.44		72.47 ± 13.07	69.81	75.13	
Unemployed	36 (20.9%)	67.46 ± 14.10	62.69	72.23		74.54 ± 13.86	69.85	79.23		77.31 ± 18.10	71.19	83.44		74.48 ± 14.28	69.65	79.31	
Length of Treatment
1–13 days (<2 weeks)	71 (41.3%)	69.67 ± 11.94	66.84	72.49	0.75	74.29 ± 10.08	71.91	76.68	0.89	80.16 ± 15.39	76.52	83.81	0.20	73.86 ± 12.92	70.80	76.92	0.85
≥ 14 days (≥2 weeks)	101 (58.7%)	69.06 ± 12.62	66.57	71.55		74.55 ± 12.61	72.06	77.03		76.98 ± 16.50	73.72	80.24		73.45 ± 14.00	70.69	76.22	
Respiratory used
No	152 (88.4%)	68.75 ± 12.08	66.81	70.68	0.10	73.63 ± 11.46	71.79	75.47	0.01*	77.63 ± 16.62	74.97	80.30	0.14	72.78 ± 13.53	70.61	74.95	0.02*
Yes	20 (11.6%)	73.57 ± 13.53	67.24	79.90		80.62 ± 11.00	75.47	85.77		83.33 ± 10.12	78.60	88.07		80.00 ± 11.97	74.40	85.60	
Length from onset (to Interview)
≤ 3 months	92 (53.5%)	67.90 ± 12.14	65.38	70.41	0.11	73.41 ± 11.32	71.07	75.76	0.21	76.54 ± 14.40	73.56	79.52	0.12	72.86 ± 13.15	70.14	75.59	0.43
>3 months	80 (46.5%)	70.94 ± 12.38	68.18	73.69		75.62 ± 11.88	72.98	78.27		80.31 ± 17.70	76.37	84.25		74.49 ± 13.98	71.38	77.61	
Long-Haul COVID 19
No	120 (69.8%)	70.12 ± 12.41	67.88	72.36	0.19	74.99 ± 11.29	72.96	77.04	0.34	78.89 ± 15.84	76.03	81.75	0.46	73.52 ± 13.68	71.04	75.99	0.88
Yes	52 (30.2%)	67.44 ± 11.99	64.10	70.78		73.16 ± 12.30	69.73	76.58		76.92 ± 16.72	72.27	81.57		73.86 ± 13.29	70.16	77.56	
Number of Long-Haul COVID 19
No	120 (69.8%)	70.12 ± 12.41	67.88	72.36	0.37	74.99 ± 11.29	72.96	77.04	0.49	78.89 ± 15.84	76.03	81.75	0.70	73.52 ± 13.68	71.04	75.99	0.45
1 Symptom	41 (23.8%)	66.99 ± 10.91	63.54	70.43		72.56 ± 11.71	68.86	76.26		76.42 ± 16.86	71.10	81.75		72.64 ± 12.61	68.66	76.62	
> 1 Symptom	11 (6.4%)	69.15 ± 15.91	58.46	79.85		75.38 ± 14.73	65.48	85.27		78.79 ± 16.82	67.49	90.09		78.41 ± 15.34	68.11	88.71	

In Table 5, the Quality-of-Life variable was categorized into two categories (good and poor), as the scores were not normally distributed. Table 5 shows female participants, those aged 40 years or more, divorced/single participants, and unemployed participants tend to have a poorer quality of life in the physical domain. People with two or more symptoms of long-haul COVID also tended to have a poor quality of life in the physical domain compared to those without long-haul symptoms (OR = 2.54, 95% CI = 0.68–9.46).

**Table 5:** The association of quality of life domain category related by sociodemographic and health-related predictors.

Variables	Frequencies (%)	Physical domain category		OR (95%CI)	Psychological domain category		OR (95%CI)	Social domain category		OR (95%CI)	Environmental domain category		OR (95%CI)
		Bad (%)	Good (%)		Bad (%)	Good (%)		Bad (%)	Good (%)		Bad (%)	Good (%)	
Gender
Male	83 (48.3%)	15 (18.1%)	68 (81.9%)	1 (Ref)	9 (10.8%)	74 (89.2%)	1 (Ref)	8 (9.6%)	75 (90.4%)	1 (Ref)	11 (13.3%)	72 (86.7%)	1 (Ref)
Female	89 (51.7%)	22 (24.7%)	67 (75.3%)	1.49 (0.71–3.11)	10 (11.2%)	79 (88.8%)	1.04 (0.40–2.70)	18 (20.2%)	71 (79.8%)	2.38 (0.97–5.81)	23 (25.8%)	66 (74.2%)	2.28 (1.03–5.04)*
Age group (Two categories)
18–39 years old	100 (58.1%)	18 (18.0%)	82 (82.0%)	1 (Ref)	7 (7.0%)	93 (93.0%)	1 (Ref)	16 (16.0%)	84 (84.0%)	1 (Ref)	21 (21.0%)	79 (79.0%)	1 (Ref)
≥ 40 years old	72 (41.9%)	19 (26.4%)	53 (73.6%)	1.63 (0.79–3.39)	12 (16.7%)	60 (83.3%)	2.66 (0.99–7.13)	10 (13.9%)	62 (86.1%)	0.85 (0.36–1.99)	13 (18.1%)	59 (81.9%)	0.83 (0.38–1.79)
Marital status
Married/Living with partner	115 (66.9%)	22 (19.1%)	93 (90.3%)	1 (Ref)	14 (12.2%)	101 (87.8%)	1 (Ref)	12 (10.4%)	103 (89.6%)	1 (Ref)	19 (16.5%)	96 (83.5%)	1 (Ref)
Divorced/Single	57 (33.1%)	15 (26.3%)	42 (73.7%)	1.51 (0.71–3.20)	5 (8.8%)	52 (91.2%)	0.69 (0.0.24–2.03)	14 (24.6%)	43 (75.4%)	2.79 (1.19–6.53)	15 (26.3%)	42 (73.7%)	1.80 (0.84–3.89)
Occupation
Civil servant & Police & army	41 (23.8%)	10 (24.4%)	31 (75.6%)	1 (Ref)	5 (12.2%)	36 (87.8%)	1 (Ref)	5 (12.2%)	36 (87.8%)	1 (Ref)	8 (19.5%)	33 (80.5%)	1 (Ref)
Private sector	95 (55.2%)	16 (16.8%)	79 (83.2%)	0.63 (0.26–1.53)	10 (10.5%)	85 (89.5%)	0.85 (0.27–2.65)	15 (15.8%)	80 (84.2%)	1.35 (0.46–3.99)	19 (20.0%)	76 (80.0%)	1.03 (0.41–2.59)
Unemployed	36 (20.9%)	11 (30.6%)	25 (69.4%)	1.36 (0.50–3.73)	4 (11.1%)	32 (88.9%)	0.90 (0.22–3.64)	6 (16.7%)	30 (83.3%)	1.44 (0.40–5.19)	7 (19.4%)	29 (80.6%)	0.99 (0.32–3.08)
Length of care/Treatment
1–13 days (<2 weeks)	71 (41.3%)	14 (19.7%)	57 (80.3%)	1 (Ref)	5 (7.0%)	66 (93.0%)	1 (Ref)	10 (14.1%)	61 (85.9%)	1 (Ref)	11 (15.5%)	60 (84.5%)	1 (Ref)
≥ 14 days (≥2 weeks)	101 (58.7%)	23 (22.8%)	78 (77.2%)	1.20 (0.57–2.53)	14 (13.9%)	87 (86.1%)	2.12 (0.73–6.19)	16 (15.8%)	85 (84.2%)	1.15 (0.49–2.70)	23 (22.8%)	78 (77.2%)	1.61 (0.73–3.56)
Respiratory used
No	152 (88.4%)	34 (22.4%)	118 (77.6%)	1 (Ref)	18 (11.8%)	134 (88.2%)	1 (Ref)	26 (17.1%)	126 (82.9%)	1 (Ref)	33 (21.7%)	119 (78.3%)	1 (Ref)
Yes	20 (11.6%)	3 (15.0%)	17 (85.0%)	0.61 (0.17–2.21)	1 (5.0%)	19 (95.0%)	0.39 (0.05–3.11)	0 (0.0%)	20 (100.0%)	-	1 (5.0%)	19 (95.0%)	0.19 (0.0.02–1.47)
Length from onset (to Interview)
≤ 3 months	92 (53.5%)	25 (27.2%)	67 (72.8%)	1 (Ref)	11 (12.0%)	81 (88.0%)	1 (Ref)	16 (17.4%)	76 (82.6%)	1 (Ref)	18 (19.6%)	74 (80.4%)	1 (Ref)
>3 months	80 (46.5%)	12 (15.0%)	68 (85.0%)	0.47 (0.22–1.02)	8 (10.0%)	72 (90.0%)	0.82 (0.31–2.15)	10 (12.5%)	70 (87.5%)	0.68 (0.29–1.59)	16 (20.0%)	64 (80.0%)	1.03 (0.48–2.18)
Long COVID
No	120 (69.8%)	22 (18.3%)	98 (81.7%)	1 (Ref)	10 (8.3%)	110 (91.7%)	1 (Ref)	15 (12.5%)	105 (87.5%)	1 (Ref)	24 (20.0%)	96 (80.0%)	1 (Ref)
Yes	52 (30.2%)	15 (28.8%)	37 (71.2%)	1.81 (0.85–3.85)	9 (17.3%)	43 (82.7%)	2.30 (0.87–6.06)	11 (21.2%)	41 (78.8%)	1.88 (0.80–4.43)	10 (19.2%)	42 (80.8%)	0.95 (0.42–2.17)
Number of long COVID
No	120 (69.8%)	22 (18.3%)	98 (81.7%)	1 (Ref)	10 (8.3%)	110 (91.7%)	1 (Ref)	15 (12.5%)	105 (87.5%)	1 (Ref)	24 (20.0%)	96 (80.0%)	1 (Ref)
1 Symptom	41 (23.8%)	11 (26.8%)	30 (73.2%)	1.63 (0.71–3.75)	7 (17.1%)	34 (82.9%)	2.26 (0.80–6.40)	9 (22.0%)	32 (78.0%)	1.97 (0.79–4.92)	8 (19.5%)	33 (80.5%)	0.97 (0.39–2.37)
> 1 Symptom	11 (6.4%)	4 (36.4%)	7 (63.6%)	2.54 (0.68–9.46)	2 (18.2%)	9 (81.8%)	2.44 (0.46–12.89)	2 (18.2%)	9 (81.8%)	1.56 (0.31–7.90)	2 (18.2%)	9 (81.8%)	0.89 (0.18–4.39)

In the psychological domain, participants aged 40 years or more and participants with 14 or more days of treatment were 2-fold more at risk of experiencing poor quality of life compared to their counterparts. Participants with two or more long-haul COVID symptoms also tended to have a higher risk for poorer quality of health in the psychological domain, with an odds ratio of 2.44 (95% CI = 0.46–12.89), compared to those who showed no symptoms.

In the social domain, people with one long-haul COVID symptom have a higher risk, by almost two times, than those with no symptoms (OR = 1.97, 95%CI = 0.79–4.92). In contradiction, long-haul COVID-19 symptoms tended to be protective against the poor quality of life compared to those with no symptoms in the environmental domain. Thus, these initial results need to be analyzed further using multivariate analysis, as seen in Table 6.

**Table 6:** Results of multivariable analysis of quality of life domains and gender, age group, marital status, occupation and having long-haul COVID 19.

No	Variables	Physical Domain	Psychological Domain	Social Domain	Environmental Domain
		AOR (95% CI)	AOR (95% CI)	AOR (95% CI)	AOR (95% CI)
1	Gender (Female/ Male)	1.50 (0.66–3.39)	1.70 (0.55–5.22)	2.30 (0.88–6.05)	2.23 (0.95–5.22)
2	Age group (≥ 40 years old/ 18–39 years old)	1.85 (0.79–4.35)	3.68 (1.17–11.61)	1.26 (0.45–3.50)	1.14 (0.46–2.80)
3	Marital status (Divorced & Single/Married & Living with partner)	1.55 (0.68–3.53)	0.80 (0.24–2.63)	2.48 (0.99–6.18)	1.63 (0.72–3.71)
4a	Occupation (Private Sector/Civil Servant & Police & Army)	0.62 (0.24–1.56)	0.94 (0.29–3.10)	1.28 (0.41–4.00)	0.98 (0.38–2.56)
4b	Occupation (Unemployed/Civil Servant & Police & Army)	0.91 (0.29–2.89)	0.55 (0.11–2.77)	0.90 (0.21–3.96)	0.70 (0.19–2.53)
5	Long-Haul COVID 19 (Yes/No)	1.93 (0.88–4.23)	2.62 (0.96–7.14)	2.09 (0.85–5.12)	0.99 (0.43–2.30)

The multivariate analysis with four models was presented in this research. Each model has one dependent variable from each domain of the Quality of Life. After adjusting with other variables, people with long-haul COVID-19 tend to be at a greater risk of having a poor quality of life in the physical domain, psychological domain, and social domain with an odds ratio of 1.93 (95% CI = 0.88–4.23), 2.62 (95% CI = 0.96–7.14), and 2.09 (95% CI = 0.85–5.12), respectively. With an odds ratio of 0.99, the effect of long-haul COVID-19 symptoms could not be concluded in the environmental domain.

## Discussion

### Long-haul COVID-19 and quality of life

Quality of life is currently defined as a multidimensional concept consisting of some domains that people consider and evaluate differently according to the importance they attach to each domain in their lives (18). In the condition of post-COVID patients, it is interesting to explore the relationship with the four domains in the WHOQOL BREF as a Quality-of-Life instrument (10). This is important to clarify the post-hospital care steps that should be taken in upholding complete health related to the quality of life.

Furthermore, based on epidemiological and immunological-based studies of recovered COVID-19 patients, it can be utilized to monitor their health status for possible future complications (2, 19). Observational investigations in larger cohorts will help us understand the deep prognosis and pathogenesis of COVID-19 disease. Studies of this kind will help uncover whether patients recovering from COVID-19 require post-acute care to recover from further infection or multi-organ damage (20). SARS-CoV-2 mainly affects people who are immunocompromised and have previous medical conditions (problems related to the lungs, kidneys, heart, and digestive tract) (11, 21). Our research showed that almost one-third (30.2%) experienced long-haul symptoms of COVID-19, with 23.8% experiencing one symptom and 6.4% experiencing two or more symptoms, in which most of them have experienced fatigue, chest pain, coughing, breathing trouble, digestive disorder, and headache (16.3, 7, 4.1, 2.9, and 2.3%), respectively.

Further, after the Quality-of-Life variable was categorized into two categories (good and poor), it was implied how the long-haul symptoms affected all domains. It was found that those with the long-haul symptoms and more than one symptom have been at approximately double the risk of having poor quality of life compared with those without symptoms. In addition, the risk of having a bad quality of life varied greatly depending on the predominance. However, the increasing risk of having poor quality of life from respirators is seen in none of the domains since most patients with respirators did not survive.

Respondents aged 40 years or over are more at risk of having poor quality of life in physical and psychological aspects of Quality of Life. In addition, women and unmarried or divorced respondents are also at risk of having poor quality of life in the physical domain. Length of stay of more than 14 days also renders poor Quality of Life in the psychological domain. In the social domain, unmarried or divorced status, as well as the type of work, seems to affect the risk of having poor quality of life. In the environmental domain, women respondents, those with single or divorced status, and those with a length of stay of more than 14 days are at risk of having a bad quality of life.

Results of multivariate analysis implied that long-haul COVID-19 patients tend to have a greater risk of having poor Quality of Life in the physical domain, psychological domain, and social domain with odds ratios of 1.93 (95% CI = 0.88–4.23), 2.62 (95% CI = 0.96–7.14), and 2.09 (95% CI = 0.85–5, 12). The environmental domain has an odd ratio of 0.99, which seems to be a protective effect for poor Quality of Life. This might be due to the items assessed for the environmental domain, such as transportation, noise, condition of living place, and access to health services, were not sensitively describing the Quality of Life of the Long-Haul COVID-19 patients.

The significant impact of the emergence of long-haul COVID-19 on recovered patients confirmed the association between long-haul COVID-19 and the WHOQOL-BREF Quality of life domains (22). The findings are essential to clarify the necessary steps for post-hospital care that need to be taken to create whole and quality-related health (23–25).

### The limitations of this research

The limitation of this research is that the symptoms reported by the subjects were obtained through online questionnaires due to the national restrictions for field data collection in 2020 until 2021. Therefore, these symptoms are subjective according to the perception of each individual. This research has a large proportion in the young age group (under 40 years) since the older age groups tend to have a higher mortality rate and did not survive after hospitalization.

Thus, persistent variation in symptoms according to age remains unknown. Notwithstanding that the patients are young and have a lower epidemiological propensity for chronic diseases and other degenerative symptoms, the symptoms of those who complain have a lower risk of the consequences of this chronic disease.

Furthermore, there were no other publications yet about quality of life using online or offline methods for long-haul COVID patients, especially in Indonesia. Thus, we could not compare our results with other studies.

### The strength of this research

Many people experience prolonged symptoms, poor health, and reduced function for months, even though they are not hospitalized for SARS-CoV-2 infection, also known as Long-Haul COVID-19. Long-Haul COVID-19 can be a serious problem for people who themselves or their families have been infected with COVID-19.

The number of studies exploring Long-Haul COVID-19 with Quality of Life at the international level were very limited, while there was no similar area at the national level. This study explores the health condition of COVID-19 patients after discharge from the hospital. Thus, the results could emphasize the need for post-hospital surveillance as a necessity, as well as building sustainable health services as a recovery response from a quality-of-life perspective.

## Conclusion

Those who have recovered from COVID-19 still have health problems, such as long-haul COVID-19 symptoms. Many of them are also related to reducing the quality of life domains. If left untreated, they will continue to experience long-term health complications. The result of this study is important for decision makers to plan and provide health services for discharged patients to maintain their quality of life. Therefore, robust post-hospital surveillance is needed to identify the need for related health services.

## Data availability statement

The original contributions presented in the study are included in the article/supplementary material; further inquiries can be directed to the corresponding author.

## Ethics statement

This study obtained ethical clearance from Universitas Indonesia’s Ethics Committee, with Ethical Approval number: 593/UN2.F10.D11/PPM.00.02/2020. The patients/participants provided their written informed consent to participate in this study.

## Author contributions

IT conceptualized and designed the study and prepared the draft of the manuscript. IT, MM, DK, and CE collected the data, contributed to the analysis, and interpreted the results. All authors contributed to the article and approved the submitted version.

## Funding

This research was supported by the International Indexed Publication Grant (PUTI) for Q2 Fiscal Year 2020 Number: NKB-1626/UN2.RST/HKP.05.00/2020 Universitas Indonesia.

## Conflict of interest

The authors declare that the research was conducted in the absence of any commercial or financial relationships that could be construed as a potential conflict of interest.

## Publisher’s note

All claims expressed in this article are solely those of the authors and do not necessarily represent those of their affiliated organizations, or those of the publisher, the editors and the reviewers. Any product that may be evaluated in this article, or claim that may be made by its manufacturer, is not guaranteed or endorsed by the publisher.

## References

[1] Logue JK Franko NM McCulloch DJ McDonald D Magedson A Wolf CR. Sequelae in adults at 6 months after COVID-19 infection. JAMA Netw Open. (2021) 4:e210830. doi: 10.1001/jamanetworkopen.2021.0830, PMID: 33606031 PMC7896197

[2] Carfì A Bernabei R Landi F. Persistent symptoms in patients after acute COVID-19. J Am Med Assoc. (2020) 324:603–5. doi: 10.1001/jama.2020.12603, PMID: 32644129 PMC7349096

[3] Davis H. E. Assaf G. S. McCorkell L. Wei H. Low R. J. Re’em Y.. Characterizing long COVID in an international cohort: 7 months of symptoms and their impact. EClinicalMedicine (2020) doi: 10.1101/2020.12.24.20248802 (Epub ahead of print). PMC8280690 34308300

[4] Huang Y. Pinto M. D. Borelli J. L. Mehrabadi M. A. Abrihim H. Dutt N.. COVID symptoms, symptom clusters, and predictors for becoming a long-hauler: looking for clarity in the haze of the pandemic. (2021) medRxiv [Preprint]. doi: 10.1101/2021.03.03.21252086 PMC9510954 36154716

[5] Niazkar HR Zibaee B Nasimi A Narjes B. The neurological manifestations of COVID-19: a review article. Neurol Sci. (2020) 41:1667–71. doi: 10.1007/s10072-020-04486-3, PMID: 32483687 PMC7262683

[6] Komaroff AL Bateman L. Will COVID-19 Lead to Myalgic encephalomyelitis/chronic fatigue syndrome? Front Med. (2021) 7:606824. doi: 10.3389/fmed.2020.606824 PMC7848220 33537329

[7] Carfi A Bernabei R Landi F Group GAC-P-ACS. Persistent symptoms in patients after acute COVID-19. JAMA . (2020) 324:603–5. doi: 10.1001/jama.2020.12603, PMID: 32644129 PMC7349096

[8] Townsend L Dyer AH Jones K Dunne J Mooney A Gaffney F. Persistent fatigue following SARS-CoV-2 infection is common and independent of severity of initial infection. PLoS One . (2020) 15:e0240784. doi: 10.1371/journal.pone.0240784, PMID: 33166287 PMC7652254

[9] Blomberg B Mohn KG-I Brokstad KA Zhou F Linchausen DW Hansen B-A. Long COVID in a prospective cohort of home-isolated patients. Nat Med. (2021) 27:1607–13. doi: 10.1038/s41591-021-01433-3 34163090 PMC8440190

[10] Skevington S Lotfy M O'Connell KA. The World Health Organization’s WHOQOL-BREF quality of life assessment: psychometric properties and results of the international field trial a report from the WHOQOL group. Qual Life Res. (2004) 13:299–310. doi: 10.1023/B:QURE.0000018486.91360.00, PMID: 15085902

[11] WHO. Division of mental health and prevention of substance abuse. WHOQOL User Manual WHO/HIS/HIS Rev. (2012) 3:1–12.

[12] The WHOQOL group. Development of the World Health Organization WHOQOL-BREF quality of life assessment. Psychol Med. (1998) 28:551–8. doi: 10.1017/s0033291798006667 9626712

[13] Nørholm V Bech P. The WHO quality of life (WHOQOL) questionnaire Danish validation study. Nord J Psychiatry. (2001) 55:229–35. doi: 10.1080/080394801681019075, PMID: 11839112

[14] Hawthorne G Herrman H Murphy B. Interpreting the WHOQOL-BRE` f: preliminary population norms and effect sizes. Soc Indic Res. (2006) 77:37–59. doi: 10.1007/s11205-005-5552-1

[15] Purba FD Hunfeld JAM Iskandarsyah A Fitriana TS Sadarjoen SS Passchier J. Quality of life of the Indonesian general population: test-retest reliability and population norms of the EQ-5D-5L and WHOQOL-BREF. PLoS One. (2018) 13:e0197098. doi: 10.1371/journal.pone.0197098, PMID: 29750806 PMC5947896

[16] Resmiya L Ifa HM. Pengembangan Alat Ukur Kualitas Hidup Indonesia. J Psikol Insight. (2019) 3:20–31. doi: 10.17509/Insight.V3i1.22247

[17] Nurbasari NA Dwipa L Gondodiputro S. The Elderly’s quality of life in the Panti Werdha and the community of Bandung City: Whoqol-Bref and Whoqol-old Indonesian version. Share Soc Work J. (2019) 9:219. doi: 10.24198/Share.V9i2.25611

[18] Theofilou P. Quality of life: definition and measurement. Eur J Psychol. (2013) 9:150–62. doi: 10.5964/ejop.v9i1.337

[19] Balachandar V Mahalaxmi I Subramaniam M Kaavya J Senthil Kumar N Laldinmawii G. Follow-up studies in COVID-19 recovered patients—is it mandatory? Sci Total Environ. (2020) 729:139021. doi: 10.1016/j.scitotenv.2020.139021, PMID: 32360909 PMC7184970

[20] Orrù G Bertelloni D Diolaiuti F Mucci F Di Giuseppe M Biella M. Long-COVID syndrome? A study on the persistence of neurological. Psychol Physiol Sympt Healthcare. (2021) 9:575. doi: 10.3390/healthcare9050575, PMID: 34068009 PMC8152255

[21] Cabrera Martimbianco AL Pacheco RL Bagattini ÂM Riera R. Frequency, signs and symptoms, and criteria adopted for long COVID-19: a systematic review. Int J Clin Pract. (2021) 75:e14357. doi: 10.1111/ijcp.14357, PMID: 33977626 PMC8236920

[22] Baig AM. Chronic COVID syndrome: Need for an appropriate medical terminology for long-COVID and COVID long-haulers. J Med Virol. (2021) 93:2555–6. doi: 10.1002/jmv.26624, PMID: 33095459

[23] Osikomaiya B Erinoso O Wright KO Odusola AO Thomas B Adeyemi O. Long COVID’: persistent COVID-19 symptoms in survivors managed in Lagos state, Nigeria. BMC Infect Dis. (2021) 21:304. doi: 10.1186/s12879-020-05716-x, PMID: 33765941 PMC7993075

[24] Greenhalgh T Knight M A’Court C Buxton M Husain L. Management of post-acute covid-19 in primary care. BMJ. (2020) 370:m3026. doi: 10.1136/bmj.m3026 32784198

[25] Al-Aly Z Xie Y Bowe B. High-dimensional characterization of post-acute sequelae of COVID-19. Nat Juli. (2021) 594:259–64. doi: 10.1038/s41586-021-03553-9, PMID: 33887749

